# The role of transcriptional regulators in metal ion homeostasis of *Mycobacterium tuberculosis*

**DOI:** 10.3389/fcimb.2024.1360880

**Published:** 2024-03-11

**Authors:** Shuxian Wang, Ren Fang, Hui Wang, Xiaotian Li, Jiayin Xing, Zhaoli Li, Ningning Song

**Affiliations:** ^1^ Key Laboratory of Respiratory Tract Pathogens and Drug Therapy, School of Life Science and Technology, Shandong Second Medical University, Weifang, China; ^2^ Drug Innovation Research Center, SAFE Pharmaceutical Technology Co. Ltd., Beijing, China

**Keywords:** *Mycobacterium tuberculosis*, transcriptional regulators, metal ions, drug development, targets

## Abstract

Metal ions are essential trace elements for all living organisms and play critical catalytic, structural, and allosteric roles in many enzymes and transcription factors. *Mycobacterium tuberculosis* (MTB), as an intracellular pathogen, is usually found in host macrophages, where the bacterium can survive and replicate. One of the reasons why Tuberculosis (TB) is so difficult to eradicate is the continuous adaptation of its pathogen. It is capable of adapting to a wide range of harsh environmental stresses, including metal ion toxicity in the host macrophages. Altering the concentration of metal ions is the common host strategy to limit MTB replication and persistence. This review mainly focuses on transcriptional regulatory proteins in MTB that are involved in the regulation of metal ions such as iron, copper and zinc. The aim is to offer novel insights and strategies for screening targets for TB treatment, as well as for the development and design of new therapeutic interventions.

## 1 Introduction

Tuberculosis (TB) is a major infectious disease caused by *Mycobacterium tuberculosis* (MTB) and poses a serious threat to global public health. According to the 2023 report of the World Health Organization, in 2022, TB was the second most common single infectious disease in the world after coronavirus disease (COVID-19), causing almost twice as many deaths as AIDS (WHO, 2023). More than 10 million people still develop TB each year (WHO, 2023). Despite the century-long fight against TB, the progress in research for its prevention and treatment has been slow, mainly due to insufficient studies of the mechanisms of its pathogenesis and latent infection.

Metal ions, including iron (Fe), zinc (Zn), copper (Cu), nickel (Ni), cobalt (Co), and manganese (Mn), are essential for a wide range of physiological processes. Their importance lies in their catalytic role in biochemical reactions as cofactors for redox, electron transfer, and hydrolase enzymes, as well as their structural allosteric effect on the structure of biomolecules (Lee et al., 2012; Sharma et al., 2023). All forms of life require trace amounts of metal ions to perform their physiological functions. It is estimated that about 30% of proteins and nearly 50% of enzymes require trace amounts of metal ions as cofactors for their life activities or structural modifications (Shafeeq et al., 2013). Metal ions play an important role not only in growth, replication, and cellular metabolism (Waldron and Robinson, 2009; Botella et al., 2012; Burcham et al., 2020) but also in the proliferation of pathogens and in the synergistic interaction between the host and the formation of antimicrobial radicals (Nairz et al., 2014).

**Figure 1:**
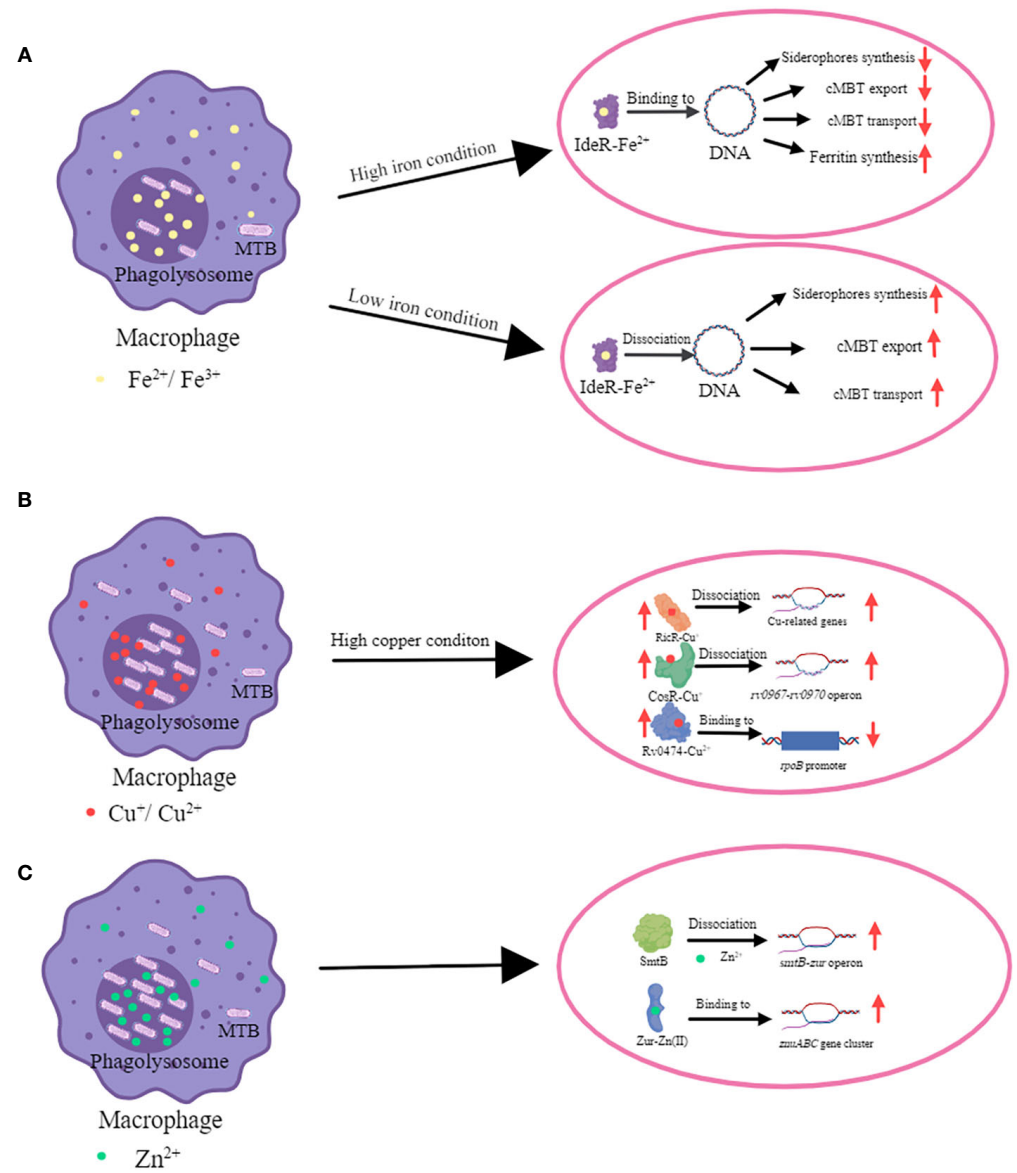
The mechanism of transcriptional regulators in metal ion homeostasis in MTB. **(A)** The mechanism of iron homeostasis regulated by IdeR. The main function of the IdeR is to repress the expression of iron uptake genes when the intracellular iron concentration is enriched, thus preventing the iron concentration from becoming too high to reach toxic levels (Bradley et al., 2020). When iron ion level is high, IdeR binds with Fe^2+^ and this complex binds to the promoter region of targeted genes which are regulated by IdeR. IdeR downregulates the expression of siderophores (include MBT and cMBT) synthases, associated transport proteins, and genes involved in iron uptake; and upregulates the expression of iron storage proteins. MTB and cMTB have an extremely high affinity for iron ions and can withdraw it from insoluble hydroxides and iron-binding proteins (Gobin and Horwitz, 1996; Rodriguez et al., 2022). MTB increases cMBT secretion to obtain more iron ions by chelating them from outside of bacteria. When iron ion level is low, IdeR dissociates from the promoters of the target genes and then the iron uptake genes are expressed (Marcos-Torres et al., 2023). **(B)** The mechanism of copper homeostasis regulated by CsoR, RicR and Rv0474c. CsoR, RicR and Rv0474 were all upregulated when copper ion is high. CsoR binds to the promoter of its own copper-sensitive operon (*cso*) containing four genes (*rv0967-rv0970*). Cu^+^-CsoR complex dissociate from *cso*, resulting in upregulation of four genes (*rv0967-rv0970*) (Liu et al., 2007). Copper also de-represses RicR from its own promoter as well as other promoters (Liu et al., 2007; Festa et al., 2011; Marcus et al., 2016). Cu^+^-RicR complex dissociates from its own promoter as well as other promoters including *mymT*, *lpqS*, *rv2963* (a putative permease gene), *socAB*, *mmcO*, and *ricR*, resulting in up-regulation of these genes (Festa et al., 2011). When Rv0474 binds with Cu^2+^, this complex can interact with *rpoB* promoter and leads to the repression of RpoB (Raghunandanan et al., 2018). **(C)** The mechanism of zinc homeostasis regulated by SmtB (Rv2358) and Zur (Rv2359). Zn^2+^ significantly weakens the affinity of SmtB for DNA, enabling RNA polymerase to load and initiate transcription of the operon (Canneva et al., 2005). Zur is activated by Zn^2+^, and it is then able to bind to the promoter region of the *znuABC* gene cluster encoding proteins which are involved in zinc uptake (Lucarelli et al., 2007).

MTB is an intracellular pathogen whose survival rate is suppressed by the mammalian immune system by affecting its metal ion homeostasis (**Figure 1**). Specifically, metal toxicity and metal starvation represent two important pathways through which the host immune system combats MTB. Host cells inhibit the growth of MTB by depriving it of essential metal ions necessary for survival, while using metal ions such as iron, copper, and zinc to inhibit MTB growth (Weiss and Schaible, 2015). An imbalance in metal ion concentration can lead to the inactivation of metalloproteins or the generation of reactive oxygen species (ROS) via the Fenton reaction, which can be toxic to cells (Eijkelkamp et al., 2015; Eom and Song, 2019). Therefore, MTB ensures its survival in the host by regulating the transport of metal ions in macrophages. The crucial part of this process involves proteins that are relevant to the regulation of metal ion concentration. These proteins act synergistically to maintain metal ion homeostasis and resistance by modulating the expression of metal ion transporter proteins.

Hence, an in-depth and systematic study of the regulatory mechanisms governing regulatory proteins is essential for elucidating the interaction between MTB, metal ions, and the host. In this review, we mainly focus on elucidating the roles of transcriptional regulatory proteins in MTB that regulate metal ions such as iron, copper and zinc, with the aim of providing ideas and new strategies for screening new targets for TB treatment and developing and designing new therapeutic interventions (**Supplementary Table S1**).

## 2 Metal ion-dependent proteins

### 2.1 Iron- dependent transcriptional regulatory proteins

Iron is essential for MTB survival and plays a crucial role in the dysregulated metabolism of TB. This metal serves as a limiting factor for MTB infection, making it challenging for the bacterium to replicate and persist in environments with low iron concentrations (Phelan et al., 2018). Conversely, an excess of intracellular iron promotes lipid peroxidation by generating reactive oxygen species (ROS) via the Fenton reaction, eliminating MTB (Chen et al., 2021).

Like almost all mammalian pathogenic bacteria, MTB must acquire iron ions from the host. Although iron is the fourth most abundant element in the earth’s crust, free iron ions are very scarce. Additionally, the concentration of iron ions in host tissues is further diminished as they can bind to iron-binding proteins like transferrin, lactoferrin and ferritin (Weinberg, 1974; Hood and Skaar, 2012; Marcela Rodriguez and Neyrolles, 2014). The host also has molecular mechanisms to inhibit bacterial iron acquisition. One such mechanism involves siderocalin (Scn), a protein that binds to MTB iron carriers capturing host iron, and preventing MTB from acquiring iron. Additionally, haptoglobin (Hpt) binds to free haemoglobin (Hb) with high affinity, thereby reducing the peripheral iron concentration in the host (Spagnuolo et al., 2011). The host tries to limit the available iron for microbial growth. To compete for iron ions, MTB has evolved a range of molecular mechanisms that target both the utilization and storage of iron ions. These adaptations are especially prominent under conditions like long-term hypoxia, low pH, and other environmental factors within the host, allowing MTB to optimize its iron acquisition strategies. MTB can produce two types of iron carriers: the fat-soluble mycobactin (MBT) and the water-soluble carboxymycobactin (cMBT)/exochelin (Gobin et al., 1995; Gobin and Horwitz, 1996; Yeruva et al., 2006; Fang et al., 2015; Tinaztepe et al., 2016; Arnold et al., 2020). MBT located in the bacterial cell membrane and cMBT is an extracellular iron carrier. The cMBT can be released externally and has the ability to obtain insoluble iron from host iron sources such as transferrin and ferritin (Macham et al., 1975; Gobin and Horwitz, 1996; Ratledge, 2004). The iron homeostasis of MTB is closely related to the oxidative stress response and must be tightly regulated because excess iron can be toxic to MTB (Imlay, 2013; Pandey and Rodriguez, 2014; Moraleda-Muñoz et al., 2019; Guth-Metzler et al., 2020).

In MTB, genes associated with iron homeostasis are tightly regulated by iron-dependent transcription factors, mainly including IdeR (Iron-dependent regulator, Rv2711), HupB (HU homologue, histone-like protein, Rv2986c), and Rv1474c (Iron-dependent regulatory protein). IdeR belongs to the DtxR family and acts as a virulence factor in MTB (Feese et al., 2001). Survival of the IdeR-deficient strains was compromised in macrophage and mouse models, underscoring the significant contribution of IdeR to the iron homeostasis, growth and virulence of MTB (Pandey and Rodriguez, 2014). IdeR consists of 230 amino acids and the first 140 amino acid residues share 88% similarity with DtxR, implying a similar function between IdeR and DtxR (Eckelt et al., 2015). The main function of the IdeR is to repress the expression of iron uptake genes when the intracellular iron concentration is enriched, thus preventing the iron concentration from becoming too high to reach toxic levels (Bradley et al., 2020). When intracellular iron levels are low, the metal-free IdeR is inactive and iron uptake genes are expressed (Marcos-Torres et al., 2023). IdeR plays a crucial role in bacterial iron homeostasis, regulating the expression of iron carrier synthases, associated transport proteins, and genes involved in iron uptake and storage (Feese et al., 2001; Gold et al., 2001; Merchant and Spatafora, 2014; Pandey and Rodriguez, 2014). IdeR also influences genes related to Fe-S clusters and metabolic enzymes (Gold et al., 2001; Pandey and Rodriguez, 2014; Deng and Zhang, 2015; Kurthkoti et al., 2015; Cheng et al., 2018; Zondervan et al., 2018; Marcos-Torres et al., 2023). While inhibiting iron uptake, IdeR activates iron storage genes, illustrating how IdeR helps bacteria respond to changes in iron availability and oxidative stress (Rodriguez et al., 2002; Pandey and Rodriguez, 2014; Kurthkoti et al., 2015). When the concentration of iron is high, IdeR binds to iron ions. This binding activates the regulator, allowing it to recognize highly conserved DNA sequences located in the promoter region of its target genes. It was found that under conditions of iron deprivation, IdeR failed to bind with the promoters of target genes. As a result, all genes related to iron uptake were induced, and the synthesis of MTB iron storage proteins was inhibited (Gold et al., 2001). IdeR also regulates mycobacteriocin synthesis by repressing the transcription of genes associated with mycobacteriocin synthesis in the presence of iron. Additionally, IdeR acts as a positive regulator for the expression of the MTB iron storage proteins, ferritin BfrA and BfrB (Gold et al., 2001). Analysis of binding site mutations and metal competition revealed that IdeR contains a high-affinity binding site for Zn^2+^, assigned to the physical metal binding site II. When Zn^2+^is present, IdeR needed a 30-fold reduction in Fe^2+^ concentration to activate DNA binding, as opposed to Fe^2+^ alone. This suggests that IdeR plays a role of a multifunctional metal repressor in conditions where Zn^2+^ serves as a structural metal and Fe^2+^ triggers physiologically relevant promoter binding.

HupB (Rv2986c), also known as MDP1 and LBP, is a cell wall-associated iron-regulated protein expressed at very low iron concentrations. HupB activates the synthesis of siderophores in MTB. When iron ion levels are lower, HupB mutant strains exhibit a significant reduction in both iron carriers and a decrease in the level of transcript levels of *mbt*, which encodes the MBT biosynthesis. The HupB-complemented strain exhibited a similar level of iron carrier production compared to that of the wild type (Pandey et al., 2014b). In contrast, IdeR was expressed in the presence of excess iron in the cell. HupB and IdeR precisely regulate siderophores biosynthesis under low and high iron concentrations, respectively (Pandey and Rodriguez, 2014; Pandey et al., 2014a; Granados-Tristán et al., 2023). HupB promotes the expression of MBT and cMBT, and it induces the Th2 immune response. However mutant strains lacking HupB fail to survive in macrophages, highlighting the crucial role of HupB in the host interactions (Pandey et al., 2014b; Choudhury et al., 2022). Moreover, the expression of HupB was detected in TB patients (Yeruva et al., 2006), and anti-HupB antibody level was found to be elevated in these patients with low serum iron levels, indicating an up-regulation of HupB expression (Sivakolundu et al., 2013). The presence of this protein *in vivo*, coupled with its essential role in countering MTB survival within the host, suggests that HupB could be a promising anti-TB vaccine candidate (Pandey et al., 2014b).

In addition, Rv1474c, a transcriptional iron-dependent regulatory protein, regulates the expression of the essential protein aconitase (Acn), which contains a [4Fe4S] cluster. Acn plays a role not only in the energy cycle but also binds to predicted iron-responsive RNA elements. Rv1474c orchestrates the iron-dependent regulation of Acn *in vivo*, when MTB is grown under iron-deficient conditions. Besides ferric ions, Rv1474c can bind tetracycline and affect its DNA-binding capability (Balakrishnan et al., 2017).

### 2.2 Copper-dependent transcriptional regulatory proteins

Copper is essential for various biochemical processes and plays a crucial role in enzymatic reactions (White et al., 2009; Stafford et al., 2013; Marcela Rodriguez and Neyrolles, 2014). As a specialized aerobic bacterium, MTB requires copper for replication (Grosse-Siestrup et al., 2021), but Copper uptake systems have not been identified in MTB (Marcela Rodriguez and Neyrolles, 2014). Copper is a well-known antimicrobial agent (Samanovic et al., 2012; Shi et al., 2014), and macrophages use it as the defensive weapon against MTB, therefore copper resistance is necessary for the virulence of MTB (Wolschendorf et al., 2011). The high concentration of copper ions effectively targets and exerts bactericidal effects on phagocytosed microorganisms (Fu et al., 2014; Darwin, 2015). In the host, MTB encounter Cu^+^ within the phagolysosomes of macrophages, and Cu^+^ can undergo Fenton chemistry *in vitro*, reacting with hydrogen peroxide to produce hydroxyl radicals (OH^-^), which in turn damages lipids, proteins and DNA (Hood and Skaar, 2012). In response to excessive levels of copper ions, bacterial pathogens adopt complex copper-sensing and detoxification mechanisms. At a transcriptional level, copper resistance operons typically follow a basic framework comprising: (a) copper-metal transcriptional regulators (usually repressors): (b) copper-binding proteins; and (c) P-type ATPases (Festa and Thiele, 2012). Among these, the copper transporter protein B (MctB) and ATPase CtpV are pivotal components. They play a crucial role in the proliferation of pathogens by mitigating copper toxicity within the host, particularly during macrophage phagocytosis, thereby limiting levels of copper ions (Wolschendorf et al., 2011; Botella et al., 2012; Hodgkinson and Petris, 2012; Neyrolles et al., 2013). Within MTB, the regulatory proteins such as CsoR (Rv0967), RicR (Rv0190), Rv0474 and SigC (Rv2069) have the capacity to modulate copper ion concentrations. CsoR, RicR and Rv0474c function at levels of copper ions that are toxic, whereas SigC functions under conditions of copper deficiency.

The copper inducible transcriptional repressor CsoR was firstly founded in MTB (Liu et al., 2007) and regulates the copper-sensitive operon (*cso*), containing four genes (*rv0967*-*rv0970*), namely *rv0967* (CsoR), *rv0968* (conserved protein), *rv0969* (a copper exporting ATPase, CtpV) and *rv0970* (probably conserved integral membrane protein). The crystal structure of CsoR indicates that the protein forms a homodimer with each monomer bound to one molar equivalent of Cu^+^ (Liu et al., 2007). The binding variant of copper reduces the binding affinity of DNA. This resulted in down-regulation of the copper-sensitive manipulator (*cso*) at the transcriptional level in response to copper stress. *Csor* (*rv0967*) and *CtpV* (*rv0969*) genes in the *cso* operon are induced by an increasing level of copper ions but not by nickel and Zinc ions (Liu et al., 2007). Transcriptome analysis of MTB cultures exposed to different copper concentrations showed that 30 copper-responsive genes in MTB changed, with half of them (including *rv0967c*) only being induced with the addition of 50 μM copper ions (Ward et al., 2008). CtpV is a putative copper exporter and is utilized by MTB to maintain resistance to copper toxicity. It is reported that a mutant strain of CtpV resulted in changes at the transcription level of 98 genes, which explains the increased stress experienced by the bacteria in the absence of this detoxification mechanism (Ward et al., 2010). Mice infected with the CtpV mutant showed reduced lung damage and immune response and a significantly longer survival time compared to wild-type (Ward et al., 2010). The study of CtpV is the evidence of a link between bacterial copper responses and MTB virulence, supporting the hypothesis that copper responses may play an important role for intracellular pathogens (Ward et al., 2010).

RicR (Rv0190) is the second copper transcriptional repressor found in MTB to defend against copper toxicity (Higgins and Giedroc, 2014; Shi et al., 2014). RicR is homologous to CsoR and regulates more genes than CsoR, including *mymT* (encodes a mycobacterial metallothionein), *lpqS* (encodes a putative lipoprotein), *rv2963* (a putative permease gene), *socAB* (small open reading frame induced by copper A and B), *mmcO* (a mycobacterial lipoprotein), and *ricR* itself (Shi et al., 2014). Disruption of RicR leads to the greater regulatory impact on copper-stressed bacteria compared to CsoR (Festa et al., 2011). It is unclear why MTB has two copper-specific sensors similar to CsoR, but this may be related to the different set points or cellular sensitivities of copper sensed by CsoR and RicR (Higgins and Giedroc, 2014). Deletion or disruption of a single RicR-regulated gene had no effect on virulence in mice. Disruption of any of the RicR-regulated genes, except *mymT*, was not sufficient to sensitize MTB to copper. It is suggested that several RicR regulated genes are required simultaneously *in vivo* to counteract copper toxicity, or the regulator is also important for resistance to host defence mechanisms that are not dependent on copper (Shi et al., 2014).

In addition, it was observed that the transcriptional regulatory protein Rv0474 in MTB can repress its own transcription under sufficient copper concentrations. Conversely, in the presence of high levels of copper ions, it stimulates its own expression, acting as a copper-responsive transcriptional regulator protein under conditions of elevated copper toxicity. Upon binding with copper, Rv0474 is directed to the promoter region of the gene encoding the RNA polymerase B subunit (RpoB). This interaction leads to the repression of both RpoB and the regulatory mechanism mediated by Rv0474. Of note, this regulatory function is specific to pathogenic *Mycobacterium* spp (Raghunandanan et al., 2018). Additionally, Rv0474 exhibits elevated expression levels during the transition from dormancy to resuscitation in MTB (Gopinath et al., 2015). It is suggested that Rv0474 could serve as a crucial target for drug design in the treatment of TB during latent infection.

Sigma factor C (SigC) has been associated with MTB virulence in various animal models. It plays a role in preventing copper starvation and serves as a transcriptional regulator of copper ion acquisition when copper is limited (Grosse-Siestrup et al., 2021). Previous studies have revealed that the growth of *sigC*-deficient strains was slower than that of the wild-type strain in a copper ion-deprived medium. Transcriptomic analysis revealed differential expression of genes controlled by the *nrp* operon and 40 additional genes under lower copper concentrations. However, supplementation with copper ions reversed the growth defects and eliminated the transcriptional differences in most of the genes. In addition, prolonged induction of SigC resulted in increased expression levels of *rv0846-rv0850*, which encode the copper-responsive regulator RicR and its operon genes. This increase suggested that prolonged induction of SigC leads to excessive copper uptake.

### 2.3 Zinc-dependent transcriptional regulatory proteins

Intracellular Zinc ions play a crucial role in the functioning of the host immune system. The significance of Zinc for the host’s immune function of host is underscored by statistics indicating an increased incidence of several infectious diseases in developing countries associated with Zinc deficiency. Adequate dietary supplementation of Zn may have a positive impact on the prevention of infectious diseases (Shafeeq et al., 2013). Zn catalyzes physiological and biochemical reactions by forming stable complexes with enzymes and proteins. Most zinc-containing enzymes play a crucial role in hydrolysis or related transfer reactions essential for cell survival. As more protein structures were analyzed, the number of zinc-containing proteins with Zn action sites in MTB significantly increased. Zn was found to be an important component of a number of enzymes, including Zn metallopeptidases, carbonic anhydrases, and fructose bisphosphate aldolases (Marcela Rodriguez and Neyrolles, 2014). Clinical studies have confirmed that Zn ions can effectively counteract MTB (Mazumder et al., 2018). In high concentrations of Zn^2+^, Zinc ions can bind to non-homologous proteins in MTB more effectively than the natural metal ions that typically bind to these proteins. This ultimately leads to protein dysfunction, effectively inhibiting the growth of MTB (Lucarelli et al., 2007).

In MTB, two key zinc regulatory proteins, SmtB (Rv2358) and Zur (Rv2359), work together to regulate the uptake or release of zinc ions. Using *M. smegmatis* (Ms) as a model system, it was found that *smtB* and *zur* genes are co-transcribed from a common promoter induced by Zn (Milano et al., 2004). The SmtB (Rv2358) protein appears to be responsible for the zinc-dependent repression of the *smtB-zur* operon. Binding of SmtB to Zn significantly weakens the DNA binding affinity, enabling RNA polymerase to load and initiate transcription of the operon (Canneva et al., 2005). Zur (zinc uptake regulator, formerly known as FurB) is a transcriptional repressor, which controls 21 genes whose expression is up-regulated under Zn^2+^ limiting conditions (Maciag et al., 2007). When MTB infects the host, intracellular zinc ions can be accumulated in macrophage phagolysosomes. High level of zinc ions are used to response MTB infection (Botella et al., 2011). There are significant fluctuations in Zn^2+^ concentration in MTB throughout the infection process. Zn^2+^ rapidly accumulates in phagosomes, but MTB has been demonstrated to counteract the toxic effects of Zn^2+^ on macrophages through the use of P1-type ATPase (Wagner et al., 2005; Botella et al., 2011).

The host utilizes the pathogen’s requirement for metal ions to generate factors that restrict the metal supply, a phenomenon referred to as trophic immunity (Zackular et al., 2015). However, this defence mechanism seems ineffective in controlling MTB infection (Dow et al., 2021). When MTB senses low concentration of Zn ions, calprotectin activates the Zn shedding regulator Zur *in vitro* (Rv2359) (Dow et al., 2021). *In vitro*, extended periods of low Zn^2+^ concentration lead to a plethora of physiological changes, such as differential expression of specific antigens, altered lipid metabolism, and different morphologies on the cell surface. In addition, Zn^2+^-restricted MTB enhances defences against oxidative stress by increasing the expression of proteins involved in DNA repair and antioxidant activity. This includes the upregulation of KatG and AhpC proteins associated with the toxicity response, along with altered utilization of redox cofactors to counteract oxidative stress (Dow et al., 2021).

### 2.4 Additional metal ion transcriptional regulatory proteins

There are other metal transcriptional regulators in MTB that regulate metal ions such as Ni, Cd and Co. Within the family of metal-sensing transcriptional regulatory proteins in MTB, the ArsR-SmtB family exhibits the greatest diversity, featuring various different metal-binding and non-metal-binding motifs. There are 12 ArsR homologues in MTB (Gao et al., 2011, 2012), and the ArsR-SmtB proteins primarily function as de-repressors, detaching from the target gene DNA upon binding to metal ions, thereby facilitating DNA transcription. Four proteins from this family, namely KmtR (Kanamycin resistance regulator, Rv0827c), CmtR (cobalt/magnesium transporter regulator, Rv1994c), SmtB (Rv2358), and NmtR (nickel-cobalt resistance regulator,Rv3744), have been successively investigated (Cavet et al., 2003; Campbell et al., 2007; Chauhan et al., 2009; Gao et al., 2011). These four proteins serve as metal-sensing entities, with KmtR and NmtR sensing nickel-cobalt, CmtR sensing cadmium and lead, and SmtB sensing Zn (Gao et al., 2012). To clarify, CmtR also senses Cd and lead (Pb), while SmtB senses Zn.

### 2.5 Other proteins and metabolites that affect metal ion concentration

#### 2.5.1 Metabolites

Recently, a novel MTB strategy for Zn acquisition has been identified. To take up Zn ions, MTB secretes and imports low-molecular-weight zinc-binding compounds named kupyaphores. This zinc-binding compound metabolite, characterized by a low molecular weight, is synthesized by a biosynthetic cluster (Rv0097-Rv0101) containing five genes in the MTB genome, with a full length of 10.8 kbp. Kupyaphores act as metal carriers. They are synthesized during the early stages of MTB infection, based on the required needs. Their properties allow them to regulate host-pathogen interactions as well as Zn imbalance. At different concentrations of Zn ions, kupyaphores secreted by MTB are tightly regulated, contributing to MTB survival under nutrient-poor and low-toxicity conditions (Mehdiratta et al., 2022). MTB lacking kupyaphores cannot store sufficient amounts of Zn ions.

#### 2.5.2 Enzymes

RiP1 hydrolase (Rv2869c), an intramembrane protein is essential for MTB replication in the mouse infection model. However, the physiological role of this regulatory system is unclear (Schneider et al., 2014; Nelson et al., 2023). Studies have shown that RiP1 induces the expression of a small RNA, which represses PdtaR-regulated proteins by sensing NO and excess copper ions. This process promotes the expression of genes associated with virulence, enhancing MTB survival during infection (Buglino et al., 2021). RiP1 demonstrates its adaptability to stress conditions by cleaving membrane-bound transcriptional regulators. RiP1 is essential for MTB growth under low iron and Zn conditions. It has been reported, and supported by transcriptomics, that RiP1 and SigL synergistically promote MTB growth under low-iron conditions. Additionally, it was demonstrated that deletion of RiP1 and SigL produced an amplified iron starvation response (Nelson et al., 2023).

#### 2.5.3 Secretory system

When bacteria infect the host, MTB can interact with host dendritic cells (DC) and macrophages (Macrophages (Mø)) through the secretion system. In this case, the secreted protein ESX regulates the immune function of host cells and mediates MTB survival of within the host. The ESX secretion system is involved in the virulence and pathogenicity of MTB, comprised of five independently functioning subsystems (ESX-1, ESX-2, ESX-3, ESX-4, and ESX-5), the ESX secretion system is the main secretion system for specific proteins in MTB. This ESX-3 secretion system plays an important role in the metabolism and growth of *Mycobacterium*, with its mutations severely inhibiting the growth of the bacteria (Gokhale and Iyer, 2022). ESX-3 is highly conserved in several *Mycobacterium* species, and is one of the VII secretion systems of proteins involved in the TB pathogenesis (Bitter et al., 2009; Stoop et al., 2012). ESX-3 secretion system is required for MBT-mediated iron acquisition (Siegrist et al., 2009), and expression of the *esx-3* operon is regulated by Zur and IdeR, to maintain the dynamic balance of Zn and iron, respectively (Rodriguez et al., 2002; Serafini et al., 2013; Kim et al., 2017; Li et al., 2020). Under high iron and Zn concentrations, metalloproteins negatively regulate ESX-3 transcription (Granados-Tristán et al., 2023). In *M. smegmatis*, ESX-3 T7SS is involved in metal dynamic homeostasis, therefore, we hypothesized that ESX-3 T7SS is closely related to the virulence of MTB. ESX-3 secretes a heterodimer consisting of EsxG (TB9.8) and EsxH (TB10.4), which disrupts phagocytic vesicle maturation in macrophages and plays an essential role in exerting toxicity in mice (Tinaztepe et al., 2016). In addition, the secretion of EsxG and EsxH is regulated by iron and Zn.

ROS are unavoidable and unfavourable factors during intracellular MTB infection. Excess ROS can induce oxidative stress and control bacterial infection by destroying essential cellular components in bacteria such as proteins, lipids, and nucleic acids (Imlay, 2003). CmtR-Zur-ESX3-Zn^2+^, a new regulatory pathway that contributes to the survival of *Mycobacterium* under oxidative stress, was identified in *M. bovis* (Li et al., 2020) The expression of CmtR (Rv1994c), a metal-sensing regulatory protein belonging to the ArsR-SmtB family is significantly induced under H_2_O_2_ stress. CmtR can interact with the negative regulatory protein, Zur, to repress the expression of the *esx-3* operon, which leads to the accumulation of intracellular Zn ions in *Mycobacterium* and thus facilitates the detoxification of ROS. Zn^2+^ serves as an effector molecule of CmtR, and CmtR can use it to repress its own expression, further facilitating antioxidant adaptations in bacteria. Similarly, CmtR can induce EsxH expression and inhibit phagosome maturation in macrophages (Li et al., 2020). In essence, CmtR is important for bacterial survival in macrophages and infected mouse lungs.

#### 2.5.4 Transporter proteins

NicT (Rv2856), a member of the NiCoT family of proteins in MTB, acts as a nickel and cobalt transporter with the capability to transport metals and antibiotics. It actively exports various drugs, and the presence of nickel may contribute to cross-resistance to specific antibiotics. Overexpression of NicT in *M. smegmatis* enhances tolerance to several antibiotics, including norfloxacin, sparfloxacin, ofloxacin, gentamicin, nalidixic acid, and isoniazid. The relatively low accumulation of norfloxacin in cells overexpressing NicT proteins suggests its involvement in the process of active exocytosis (Adhikary et al., 2022). NicT may serve as a promising target for new drugs or vaccines, offering potential for the development of novel therapies against drug-resistant TB.

## 3 Discussion

To meet the World Health Organization’s goal of ending the global TB epidemic by 2030, there is an urgent need for the development of new chemotherapeutic agents and safe, effective vaccines. The emergence of multidrug-resistant TB has led to more complex and challenging TB treatment regimens. In the past 40 years, only three new drugs for TB, pretomanid, delamanid, and bedaquiline, have been introduced to the market (Kundu and Basu, 2021). Research on antimicrobial therapeutics targeting metal ions in MTB is promoted due to the increasing number and variety of drug-resistant bacteria and the scarcity of new drug options. Transcription regulatory proteins in MTB that control vital metal ions associated with virulence represent promising targets for antimicrobial drug development (Yang et al., 2022). Therefore, we summarized the targets in the regulation of metal ions and provide an overview of recent advances in antituberculosis drug discovery against metal ions in MTB.

Metals such as iron, copper, and Zinc play crucial roles in key metabolic processes, pathogenicity, and proliferation of MTB, as well as in defence against host-mediated free radical formation (Nairz et al., 2014). Metal-dependent transcriptional regulatory proteins can quickly respond to varying metal concentrations in the host, allowing the microorganism to adapt to harsh conditions of the host’s environment and exploiting the host environment for their own proliferation and survival. Understanding the transcriptional regulatory network of MTB is crucial to elucidate its pathogenesis and survival mechanisms within the host. This knowledge may be a fundamental source of theoretical insight into the pathogenesis and treatment of TB.

Gaining a deeper understanding of the metal-ions-dependent stress encountered by bacteria during infection, along with the molecular mechanisms used by them to adapt to the encountered stressors paves the way for the development of the most effective drugs. Metallotoxicity and metal chelation can be used as effective means of controlling bacterial growth. Both these phenomena hold significant promise, especially in managing drug-resistant bacterial strains in clinical settings. Elucidating metal-responsive regulatory proteins from different pathogenic bacteria, along with their target genes and the factors influencing their metal specificity, will guide future TB control strategies and enhance current metal-based therapeutic approaches. Unraveling the mechanisms involved in metal utilization and access may unveil new drug or vaccine targets, unlocking pathways for innovative therapies against infections and diseases. Deciphering these mechanisms is critical to understanding how MTB adapts to metal stress at the host-pathogen interface and its role in pathogenesis.

IdeR, as an essential gene in MTB, is a very promising drug target. Inhibition of IdeR leads to fatal iron toxicity in MTB and increased sensitivity to antibiotics (Rodriguez et al., 2022). Recent research efforts have shifted towards drug development, with a significant focus on IdeR. Biochemical studies, including the characterization based on the determination of the crystal structure of IdeR and the kinetics of iron binding to DNA, have established a robust foundation for undertaking structure-based development of anti-IdeR drugs. In 2017, Rohilla et al. conducted a virtual screening against the DNA-binding domain of IdeR, which was screened against the NCI database and investigated using gel mobility shift assay (EMSA) (Rohilla et al., 2017). The results identified nine molecules with inhibitory effects on the activity of IdeR. Resolving the co-crystal structures of these small molecule inhibitors with IdeR will aid in further analyzing the key amino acid sites where IdeR exerts its important functions. This will contribute to the development of more effective inhibitors in the future. Inhibitors that restrict the survival of MTB in host macrophages have been screened in a high-throughput manner. It is reported that the compound sAELO57 acts as an iron chelator, limiting the access of MTB to iron and exhibiting activity against intracellular bacteria. sAELO57 showed enhanced inhibition of MTB growth under conditions where cholesterol was the main carbon source. The growth rate of the IdeR knockout strain was slowed down, and this effect was further reduced by the presence of sAEL057 (Theriault et al., 2022). Targeting transcription factors poses a challenge due to the necessity of disrupting intricate protein-protein or protein-DNA interactions. However, since IdeR dimerization and DNA binding rely on metal binding, blocking this metal interaction could be an effective strategy to inhibit IdeR function (Wisedchaisri et al., 2007; Rodriguez et al., 2022).Conducting high-throughput screening of natural or synthetic inhibitors is needed to exploit the apparent sensitivity of MTB to iron dysregulation (Rodriguez et al., 2022).

HupB(Rv2986c) is a lucrative but under-explored target. The crystalline structure of HupB has been investigated (Bhowmick et al., 2014). This may encourage researchers to perform high-throughput screening (HTS)-based drug discovery, scaffold jumping, and hit identification. Shyam et al. have proposed two research directions, the first of which is aimed at accelerating drug discovery through the use of zinc databases for scaffold jumping and drug repurposing. The second is to assess the potential of mycobactin biosynthesis inhibitors to interact with HupB proteins (Shyam et al., 2022). Because Rv1474c lacks homologues in the human host, it holds promise as a potential drug target for the designing novel anti-mycobacterial drugs (Balakrishnan et al., 2017).

The prolonged latent infection of MTB, has led to the emergence of multi-drug-resistant strains, presenting a significant challenge in the treatment, prevention, and control of TB due to extensive antibiotic use. Understanding the modulated immunometabolic pathways of the host following infection is crucial for the development of new therapies. Current research focuses on the development of host-directed therapies (HDT) combined with existing antimicrobials, aiming to enhance host defence. The pathophysiology of TB is closely linked to iron metabolism, a vital factor for MTB survival. Phelan (Phelan et al., 2018) et al. concluded that iron chelators, beyond their direct impact on iron utilization, have a multifaceted influence on immune metabolic function by regulating iron supply to enhance the immune response of the host to TB infection. This suggests their potential as a strategy for host-directed therapy (HDT). Additionally, ferritin, a crucial regulator of macrophage iron homeostasis, enhances host defence against MTB infection. Nuclear receptor coactivator 4 (NCOA4) is a cargo receptor identified in recent years for inducing ferritin degradation. MTB infection promotes NCOA4-mediated ferritin degradation in macrophages. This process increases the intracellular MTB bioavailability of iron, consequently promoting bacterial growth. MTB infection enhances NCOA4-mediated ferritin degradation through p38/AKT1 and TRIM21-mediated proteasomal degradation of HERC2, the E3 ligase of NCOA4. In a mouse model, NCOA4 deficiency in myeloid cells accelerated clearance of MTB. Taken together, this finding reveals a strategy by which MTB manipulates harnesses host ferritin metabolism for its own intracellular survival, highlighting a potential target for host-directed treatment of TB (Dai et al., 2023).

In addition to existing vaccines and antibiotics, metallodrugs have attracted great interest from the pharmaceutical industry. This interest is primarily attributed to their irreversible binding and antimicrobial activity on the active centers of specific enzymes, which may play an important role in the pathogenesis of TB. Combinatorial chemistry has become an important tool for the development of new approaches against MTB. Coelho et al. evaluated the anti-mycobacterial activity of phenylhydroxyformate complexes associated with two ligand metals, Cu^2+^ and Co^2+^. They found that both compounds exhibited intracellular inhibitory activity in the intracellular environment of macrophages (Coelho et al., 2020).At a concentration of 200 µg/mL, no cytotoxicity was observed, and the effect was comparable to that of rifampicin. The complexes exhibited the ability to inhibit MTB growth, even in the persistent infection phase. The potential interaction of the metal complexes with urease, an enzyme crucial for bacterial survival in a phagocytic environment *in vivo*, underscores the prospect of these metals in the development of novel anti-tuberculosis drugs.

Since MTB has evolved a set of molecular mechanisms of protection against Zn^2+^ toxicity, the use of metal-based therapies to combat MTB requires unique delivery methods or novel materials. Zn and other metal ion acquisition systems can act as protective antigens, offering opportunities for the development of novel vaccines and therapeutic approaches (Shafeeq et al., 2013). *In vitro* and *in vivo* studies have shown that Zn oxide nanoparticles (ZnONPs) can kill MTB and MTB-infected macrophages with relatively low cytotoxicity to host cells (Taranath and Patil, 2016; Yaghubi Kalurazi and Jafari, 2021; Behzad et al., 2022; Geng et al., 2023).

In conclusion, it is urgent to meet the persistent and need for TB therapeutics by continuously exploring new drug targets and developing new compounds. In the post-genomic era, advanced genetic tools, innovative molecular biology techniques, and a deeper understanding of the interactions between pathogens and host immunity will undoubtedly facilitate the discovery of new biological targets for chemotherapy, as well as the development of small-molecule drugs.

## Author contributions

SW: Writing – original draft, Writing – review & editing, Conceptualization, Data curation, Formal analysis, Validation. RF: Validation, Writing – review & editing. HW: Validation, Writing – review & editing. XL: Validation, Writing – review & editing. JX: Validation, Writing – review & editing. ZL: Validation, Writing – review & editing, Supervision, Writing – original draft. NS: Conceptualization, Data curation, Funding acquisition, Investigation, Supervision, Validation, Writing – original draft, Writing – review & editing.

## References

[B1] Adhikary A. Biswal S. Chatterjee D. Ghosh A. S. (2022). A NiCoT family metal transporter of *Mycobacterium tuberculosis* (Rv2856/NicT) behaves as a drug efflux pump that facilitates cross-resistance to antibiotics. Microbiol. (Reading) 168:001260. doi: 10.1099/mic.0.001260 36282241

[B2] Arnold F. M. Weber M. S. Gonda I. Gallenito M. J. Adenau S. Egloff P.. (2020). The ABC exporter IrtAB imports and reduces mycobacterial siderophores. Nature 580, 413–417. doi: 10.1038/s41586-020-2136-9 32296173 PMC7170716

[B3] Balakrishnan K. Mohareer K. Banerjee S. (2017). *Mycobacterium tuberculosis* Rv1474c is a TetR-like transcriptional repressor that regulates aconitase, an essential enzyme and RNA-binding protein, in an iron-responsive manner. Tuberculosis (Edinb) 103, 71–82. doi: 10.1016/j.tube.2017.01.003 28237036

[B4] Behzad F. Sefidgar E. Samadi A. Lin W. Pouladi I. Pi J. (2022). An overview of zinc oxide nanoparticles produced by plant extracts for anti-tuberculosis treatments. Curr. Med. Chem. 29, 86–98. doi: 10.2174/0929867328666210614122109 34126883

[B5] Bhowmick T. Ghosh S. Dixit K. Ganesan V. Ramagopal U. A. Dey D.. (2014). Targeting *Mycobacterium tuberculosis* nucleoid-associated protein HU with structure-based inhibitors. Nat. Commun. 5, 4124. doi: 10.1038/ncomms5124 24916461

[B6] Bitter W. Houben E. N. Bottai D. Brodin P. Brown E. J. Cox J. S.. (2009). Systematic genetic nomenclature for type VII secretion systems. PloS Pathog. 5, e1000507. doi: 10.1371/journal.ppat.1000507 19876390 PMC2763215

[B7] Botella H. Peyron P. Levillain F. Poincloux R. Poquet Y. Brandli I.. (2011). Mycobacterial p(1)-type ATPases mediate resistance to zinc poisoning in human macrophages. Cell Host Microbe 10, 248–259. doi: 10.1016/j.chom.2011.08.006 21925112 PMC3221041

[B8] Botella H. Stadthagen G. Lugo-Villarino G. de Chastellier C. Neyrolles O. (2012). Metallobiology of host-pathogen interactions: an intoxicating new insight. Trends Microbiol. 20, 106–112. doi: 10.1016/j.tim.2012.01.005 22305804

[B9] Bradley J. M. Svistunenko D. A. Wilson M. T. Hemmings A. M. Moore G. R. Le Brun N. E. (2020). Bacterial iron detoxification at the molecular level. J Biol Chem 295(51), 17602–17623. doi: 10.1074/jbc.REV120.007746 PMC7762939 33454001

[B10] Buglino J. A. Sankhe G. D. Lazar N. Bean J. M. Glickman M. S. (2021). Integrated sensing of host stresses by inhibition of a cytoplasmic two-component system controls *M. tuberculosis* acute lung infection. Elife 10:e65351. doi: 10.7554/eLife.65351 34003742 PMC8131098

[B11] Burcham L. R. Le Breton Y. Radin J. N. Spencer B. L. Deng L. Hiron A.. (2020). Identification of zinc-dependent mechanisms used by group B streptococcus to overcome calprotectin-mediated stress. mBio 11:e02302-20. doi: 10.1128/mBio.02302-20 33173000 PMC7667036

[B12] Campbell D. R. Chapman K. E. Waldron K. J. Tottey S. Kendall S. Cavallaro G.. (2007). Mycobacterial cells have dual nickel-cobalt sensors: sequence relationships and metal sites of metal-responsive repressors are not congruent. J. Biol. Chem. 282, 32298–32310. doi: 10.1074/jbc.M703451200 17726022 PMC3145109

[B13] Canneva F. Branzoni M. Riccardi G. Provvedi R. Milano A. (2005). Rv2358 and FurB: two transcriptional regulators from *Mycobacterium tuberculosis* which respond to zinc. J. Bacteriol 187, 5837–5840. doi: 10.1128/JB.187.16.5837-5840.2005 16077132 PMC1196093

[B14] Cavet J. S. Graham A. I. Meng W. Robinson N. J. (2003). A cadmium-lead-sensing ArsR-SmtB repressor with novel sensory sites. Complementary metal discrimination by NmtR AND CmtR in a common cytosol. J. Biol. Chem. 278, 44560–44566. doi: 10.1074/jbc.M307877200 12939264

[B15] Chauhan S. Kumar A. Singhal A. Tyagi J. S. Krishna Prasad H. (2009). CmtR, a cadmium-sensing ArsR-SmtB repressor, cooperatively interacts with multiple operator sites to autorepress its transcription in *Mycobacterium tuberculosis* . FEBS J. 276, 3428–3439. doi: 10.1111/j.1742-4658.2009.07066.x 19456862

[B16] Chen X. Kang R. Kroemer G. Tang D. (2021). Broadening horizons: the role of ferroptosis in cancer. Nat. Rev. Clin. Oncol. 18, 280–296. doi: 10.1038/s41571-020-00462-0 33514910

[B17] Cheng Y. Yang R. Lyu M. Wang S. Liu X. Wen Y.. (2018). IdeR, a dtxR family iron response regulator, controls iron homeostasis, morphological differentiation, secondary metabolism, and the oxidative stress response in *Streptomyces avermitilis* . Appl. Environ. Microbiol. 84:e01503-18. doi: 10.1128/AEM.01503-18 30194099 PMC6210122

[B18] Choudhury M. Virivinti J. Kandi S. Sritharan V. Sritharan M. (2022). Th2 immune response by the iron-regulated protein HupB of *Mycobacterium tuberculosis* . Indian J. Tuberc 69, 90–99. doi: 10.1016/j.ijtb.2021.04.011 35074158

[B19] Coelho T. S. Halicki P. C. B. Silva L. Jr. de Menezes Vicenti J. R. Gonçalves B. L. Almeida da Silva P. E.. (2020). Metal-based antimicrobial strategies against intramacrophage *Mycobacterium tuberculosis* . Lett. Appl. Microbiol. 71, 146–153. doi: 10.1111/lam.13298 32286695

[B20] Dai Y. Zhu C. Xiao W. Huang K. Wang X. Shi C.. (2023). *Mycobacterium tuberculosis* hijacks host TRIM21- and NCOA4-dependent ferritinophagy to enhance intracellular growth. J. Clin. Invest. 133:e159941. doi: 10.1172/JCI159941 37066876 PMC10104892

[B21] Darwin K. H. (2015). *Mycobacterium tuberculosis* and Copper: A Newly Appreciated Defense against an Old Foe? J. Biol. Chem. 290, 18962–18966. doi: 10.1074/jbc.R115.640193 26055711 PMC4521017

[B22] Deng Y. Zhang X. (2015). DtxR, an iron-dependent transcriptional repressor that regulates the expression of siderophore gene clusters in *Thermobifida fusca* . FEMS Microbiol. Lett. 362, 1–6. doi: 10.1093/femsle/fnu053 25673661

[B23] Dow A. Sule P. O'Donnell T. J. Burger A. Mattila J. T. Antonio B.. (2021). Zinc limitation triggers anticipatory adaptations in *Mycobacterium tuberculosis* . PloS Pathog. 17, e1009570. doi: 10.1371/journal.ppat.1009570 33989345 PMC8121289

[B24] Eckelt E. Meißner T. Meens J. Laarmann K. Nerlich A. Jarek M.. (2015). FurA contributes to the oxidative stress response regulation of Mycobacterium avium ssp. paratuberculosis. Front. Microbiol. 6. doi: 10.3389/fmicb.2015.00016 PMC4319475 25705205

[B25] Eijkelkamp B. A. McDevitt C. A. Kitten T. (2015). Manganese uptake and streptococcal virulence. Biometals 28, 491–508. doi: 10.1007/s10534-015-9826-z 25652937 PMC5800397

[B26] Eom H. Song W. J. (2019). Emergence of metal selectivity and promiscuity in metalloenzymes. J. Biol. Inorg Chem. 24, 517–531. doi: 10.1007/s00775-019-01667-0 31115763

[B27] Fang Z. Sampson S. L. Warren R. M. Gey van Pittius N. C. Newton-Foot M. (2015). Iron acquisition strategies in mycobacteria. Tuberculosis (Edinb) 95, 123–130. doi: 10.1016/j.tube.2015.01.004 25636179

[B28] Feese M. D. Ingason B. P. Goranson-Siekierke J. Holmes R. K. Hol W. G. (2001). Crystal structure of the iron-dependent regulator from *Mycobacterium tuberculosis* at 2.0-A resolution reveals the Src homology domain 3-like fold and metal binding function of the third domain. J. Biol. Chem. 276, 5959–5966. doi: 10.1074/jbc.M007531200 11053439

[B29] Festa R. A. Jones M. B. Butler-Wu S. Sinsimer D. Gerads R. Bishai W. R.. (2011). A novel copper-responsive regulon in *Mycobacterium tuberculosis* . Mol. Microbiol. 79, 133–148. doi: 10.1111/j.1365-2958.2010.07431.x 21166899 PMC3052634

[B30] Festa R. A. Thiele D. J. (2012). Copper at the front line of the host-pathogen battle. PloS Pathog. 8, e1002887. doi: 10.1371/journal.ppat.1002887 23028306 PMC3447745

[B31] Fu Y. Chang F. M. Giedroc D. P. (2014). Copper transport and trafficking at the host-bacterial pathogen interface. Acc Chem. Res. 47, 3605–3613. doi: 10.1021/ar500300n 25310275 PMC4268108

[B32] Gao C. H. Yang M. He Z. G. (2011). An ArsR-like transcriptional factor recognizes a conserved sequence motif and positively regulates the expression of *phoP* in mycobacteria. Biochem. Biophys. Res. Commun. 411, 726–731. doi: 10.1016/j.bbrc.2011.07.014 21782791

[B33] Gao C. H. Yang M. He Z. G. (2012). Characterization of a novel ArsR-like regulator encoded by Rv2034 in *Mycobacterium tuberculosis* . PloS One 7, e36255. doi: 10.1371/journal.pone.0036255 22558408 PMC3338718

[B34] Geng S. Hao P. Wang D. Zhong P. Tian F. Zhang R.. (2023). Zinc oxide nanoparticles have biphasic roles on *Mycobacterium*-induced inflammation by activating autophagy and ferroptosis mechanisms in infected macrophages. Microb. Pathog. 180, 106132. doi: 10.1016/j.micpath.2023.106132 37201638

[B35] Gobin J. Horwitz M. A. (1996). Exochelins of *Mycobacterium tuberculosis* remove iron from human iron-binding proteins and donate iron to mycobactins in the *M. tuberculosis* cell wall. J. Exp. Med. 183, 1527–1532. doi: 10.1084/jem.183.4.1527 8666910 PMC2192514

[B36] Gobin J. Moore C. H. Reeve J. R. Jr. Wong D. K. Gibson B. W. Horwitz M. A. (1995). Iron acquisition by *Mycobacterium tuberculosis*: isolation and characterization of a family of iron-binding exochelins. Proc. Natl. Acad. Sci. U.S.A. 92, 5189–5193. doi: 10.1073/pnas.92.11.5189 7761471 PMC41874

[B37] Gokhale K. M. Iyer A. M. (2022). Deployment of iron uptake machineries as targets against drug resistant strains of *mycobacterium tuberculosis* . Indian J. Pharmacol. 54, 353–363. doi: 10.4103/ijp.IJP_667_20 36537405 PMC9846915

[B38] Gold B. Rodriguez G. M. Marras S. A. Pentecost M. Smith I. (2001). The *Mycobacterium tuberculosis* IdeR is a dual functional regulator that controls transcription of genes involved in iron acquisition, iron storage and survival in macrophages. Mol. Microbiol. 42, 851–865. doi: 10.1046/j.1365-2958.2001.02684.x 11722747

[B39] Gopinath V. Raghunandanan S. Gomez R. L. Jose L. Surendran A. Ramachandran R.. (2015). Profiling the Proteome of *Mycobacterium tuberculosis* during Dormancy and Reactivation. Mol. Cell Proteomics 14, 2160–2176. doi: 10.1074/mcp.M115.051151 26025969 PMC4528245

[B40] Granados-Tristán A. L. Hernández-Luna C. E. González-Escalante L. A. Camacho-Moll M. E. Silva-Ramírez B. Bermúdez de León M.. (2023). ESX-3 secretion system in *Mycobacterium*: An overview. Biochimie 216, 46–55. doi: 10.1016/j.biochi.2023.10.013 37879428

[B41] Grosse-Siestrup B. T. Gupta T. Helms S. Tucker S. L. Voskuil M. I. Quinn F. D.. (2021). A role for *Mycobacterium tuberculosis* sigma factor C in copper nutritional immunity. Int. J. Mol. Sci. 22:2118. doi: 10.3390/ijms22042118 33672733 PMC7924339

[B42] Guth-Metzler R. Bray M. S. Frenkel-Pinter M. Suttapitugsakul S. Montllor-Albalate C. Bowman J. C.. (2020). Cutting in-line with iron: ribosomal function and non-oxidative RNA cleavage. Nucleic Acids Res. 48, 8663–8674. doi: 10.1093/nar/gkaa586 32663277 PMC7470983

[B43] Higgins K. A. Giedroc D. (2014). Insights into protein allostery in the csoR/rcnR family of transcriptional repressors. Chem. Lett. 43, 20–25. doi: 10.1246/cl.130965 24695963 PMC3970791

[B44] Hodgkinson V. Petris M. J. (2012). Copper homeostasis at the host-pathogen interface. J. Biol. Chem. 287, 13549–13555. doi: 10.1074/jbc.R111.316406 22389498 PMC3340201

[B45] Hood M. I. Skaar E. P. (2012). Nutritional immunity: transition metals at the pathogen-host interface. Nat. Rev. Microbiol. 10, 525–537. doi: 10.1038/nrmicro2836 22796883 PMC3875331

[B46] Imlay J. A. (2003). Pathways of oxidative damage. Annu. Rev. Microbiol. 57, 395–418. doi: 10.1146/annurev.micro.57.030502.090938 14527285

[B47] Imlay J. A. (2013). The molecular mechanisms and physiological consequences of oxidative stress: lessons from a model bacterium. Nat. Rev. Microbiol. 11, 443–454. doi: 10.1038/nrmicro3032 23712352 PMC4018742

[B48] Kim Y. S. Yang C. S. Nguyen L. T. Kim J. K. Jin H. S. Choe J. H.. (2017). *Mycobacterium abscessus* ESX-3 plays an important role in host inflammatory and pathological responses during infection. Microbes Infect. 19, 5–17. doi: 10.1016/j.micinf.2016.09.001 27637463

[B49] Kundu M. Basu J. (2021). Applications of transcriptomics and proteomics for understanding dormancy and resuscitation in *Mycobacterium tuberculosis* . Front. Microbiol. 12. doi: 10.3389/fmicb.2021.642487 PMC8044303 33868200

[B50] Kurthkoti K. Tare P. Paitchowdhury R. Gowthami V. N. Garcia M. J. Colangeli R.. (2015). The mycobacterial iron-dependent regulator IdeR induces *ferritin (bfrB)* by alleviating Lsr2 repression. Mol. Microbiol. 98, 864–877. doi: 10.1111/mmi.13166 26268801 PMC4879814

[B51] Lee C. W. Chakravorty D. K. Chang F. M. Reyes-Caballero H. Ye Y. Merz K. M. Jr.. (2012). Solution structure of *Mycobacterium tuberculosis* NmtR in the apo state: insights into Ni(II)-mediated allostery. Biochemistry 51, 2619–2629. doi: 10.1021/bi3001402 22394357 PMC3500661

[B52] Li X. Chen L. Liao J. Hui J. Li W. He Z. G. (2020). A novel stress-inducible CmtR-ESX3-Zn(2+) regulatory pathway essential for survival of *Mycobacterium bovis* under oxidative stress. J. Biol. Chem. 295, 17083–17099. doi: 10.1074/jbc.RA120.013017 33033071 PMC7863910

[B53] Liu T. Ramesh A. Ma Z. Ward S. K. Zhang L. George G. N.. (2007). CsoR is a novel *Mycobacterium tuberculosis* copper-sensing transcriptional regulator. Nat. Chem. Biol. 3, 60–68. doi: 10.1038/nchembio844 17143269

[B54] Lucarelli D. Russo S. Garman E. Milano A. Meyer-Klaucke W. Pohl E. (2007). Crystal structure and function of the zinc uptake regulator FurB from *Mycobacterium tuberculosis* . J. Biol. Chem. 282, 9914–9922. doi: 10.1074/jbc.M609974200 17213192

[B55] Macham L. P. Ratledge C. Nocton J. C. (1975). Extracellular iron acquisition by mycobacteria: role of the exochelins and evidence against the participation of mycobactin. Infect. Immun. 12, 1242–1251. doi: 10.1128/iai.12.6.1242-1251.1975 1107222 PMC415427

[B56] Maciag A. Dainese E. Rodriguez G. M. Milano A. Provvedi R. Pasca M. R.. (2007). Global analysis of the *Mycobacterium tuberculosis* Zur (FurB) regulon. J. Bacteriol 189, 730–740. doi: 10.1128/JB.01190-06 17098899 PMC1797298

[B57] Marcela Rodriguez G. Neyrolles O. (2014). Metallobiology of tuberculosis. Microbiol. Spectr. 2. doi: 10.1128/microbiolspec.MGM2-0012-2013 PMC5180607 26103977

[B58] Marcos-Torres F. J. Juniar L. Griese J. J. (2023). The molecular mechanisms of the bacterial iron sensor IdeR. Biochem. Soc. Trans. 51, 1319–1329. doi: 10.1042/BST20221539 37140254 PMC10317159

[B59] Marcus S. A. Sidiropoulos S. W. Steinberg H. Talaat A. M. (2016). CsoR Is Essential for Maintaining Copper Homeostasis in Mycobacterium tuberculosis. PLoS One 11(3), e0151816. doi: 10.1371/journal.pone.0151816 26999439 PMC4801387

[B60] Mazumder M. K. Rahim M. A. Ahmed S. Uddin M. J. Khatoon M. Patwary M. S.. (2018). Serum zinc concentrations in patients with pulmonary tuberculosis. Mymensingh Med. J. 27, 536–543. 30141443

[B61] Mehdiratta K. Singh S. Sharma S. Bhosale R. S. Choudhury R. Masal D. P.. (2022). Kupyaphores are zinc homeostatic metallophores required for colonization of *Mycobacterium tuberculosis* . Proc. Natl. Acad. Sci. U.S.A. 119:e2110293119. doi: 10.1073/pnas.2110293119 35193957 PMC8872721

[B62] Merchant A. T. Spatafora G. A. (2014). A role for the DtxR family of metalloregulators in gram-positive pathogenesis. Mol. Oral. Microbiol. 29, 1–10. doi: 10.1111/omi.12039 24034418 PMC3866218

[B63] Milano A. Branzoni M. Canneva F. Profumo A. Riccardi G. (2004). The *Mycobacterium tuberculosis Rv2358-furB* operon is induced by zinc. Res. Microbiol. 155, 192–200. doi: 10.1016/j.resmic.2003.11.009 15059632

[B64] Moraleda-Muñoz A. Marcos-Torres F. J. Pérez J. Muñoz-Dorado J. (2019). Metal-responsive RNA polymerase extracytoplasmic function (ECF) sigma factors. Mol. Microbiol. 112, 385–398. doi: 10.1111/mmi.14328 31187912 PMC6851896

[B65] Nairz M. Haschka D. Demetz E. Weiss G. (2014). Iron at the interface of immunity and infection. Front. Pharmacol. 5. doi: 10.3389/fphar.2014.00152 PMC4100575 25076907

[B66] Nelson S. J. Williams J. T. Buglino J. A. Nambi S. Lojek L. J. Glickman M. S.. (2023). The Rip1 intramembrane protease contributes to iron and zinc homeostasis in *Mycobacterium tuberculosis* . mSphere 8, e0038922. doi: 10.1128/msphere.00389-22 37318217 PMC10449499

[B67] Neyrolles O. Mintz E. Catty P. (2013). Zinc and copper toxicity in host defense against pathogens: *Mycobacterium tuberculosis* as a model example of an emerging paradigm. Front. Cell Infect. Microbiol. 3. doi: 10.3389/fcimb.2013.00089 PMC3841717 24350063

[B68] Pandey R. Rodriguez G. M. (2014). IdeR is required for iron homeostasis and virulence in *Mycobacterium tuberculosis* . Mol. Microbiol. 91, 98–109. doi: 10.1111/mmi.12441 24205844 PMC3902104

[B69] Pandey S. D. Choudhury M. Sritharan M. (2014a). Transcriptional regulation of *Mycobacterium tuberculosis hupB* gene expression. Microbiol. (Reading) 160, 1637–1647. doi: 10.1099/mic.0.079640-0 24858079

[B70] Pandey S. D. Choudhury M. Yousuf S. Wheeler P. R. Gordon S. V. Ranjan A.. (2014b). Iron-regulated protein HupB of *Mycobacterium tuberculosis* positively regulates siderophore biosynthesis and is essential for growth in macrophages. J. Bacteriol 196, 1853–1865. doi: 10.1128/JB.01483-13 24610707 PMC4010995

[B71] Phelan J. J. Basdeo S. A. Tazoll S. C. McGivern S. Saborido J. R. Keane J. (2018). Modulating iron for metabolic support of TB host defense. Front. Immunol. 9. doi: 10.3389/fimmu.2018.02296 PMC6196273 30374347

[B72] Raghunandanan S. Ramachandran R. Gomez R. L. Devanarayanan S. Bommakanti A. Kondapi A. K.. (2018). Rv0474 is a copper-responsive transcriptional regulator that negatively regulates expression of RNA polymerase β subunit in *Mycobacterium tuberculosis* . FEBS J. 285, 3849–3869. doi: 10.1111/febs.14637 30120904

[B73] Ratledge C. (2004). Iron, mycobacteria and tuberculosis. Tuberculosis (Edinb) 84, 110–130. doi: 10.1016/j.tube.2003.08.012 14670352

[B74] Rodriguez G. M. Sharma N. Biswas A. Sharma N. (2022). The iron response of *mycobacterium tuberculosis* and its implications for tuberculosis pathogenesis and novel therapeutics. Front. Cell Infect. Microbiol. 12. doi: 10.3389/fcimb.2022.876667 PMC9132128 35646739

[B75] Rodriguez G. M. Voskuil M. I. Gold B. Schoolnik G. K. Smith I. (2002). *ideR*, An essential gene in *mycobacterium tuberculosis*: role of IdeR in iron-dependent gene expression, iron metabolism, and oxidative stress response. Infect. Immun. 70, 3371–3381. doi: 10.1128/IAI.70.7.3371-3381.2002 12065475 PMC128082

[B76] Rohilla A. Khare G. Tyagi A. K. (2017). Virtual Screening, pharmacophore development and structure based similarity search to identify inhibitors against IdeR, a transcription factor of *Mycobacterium tuberculosis* . Sci. Rep. 7, 4653. doi: 10.1038/s41598-017-04748-9 28680150 PMC5498548

[B77] Samanovic M. I. Ding C. Thiele D. J. Darwin K. H. (2012). Copper in microbial pathogenesis: meddling with the metal. Cell Host Microbe 11, 106–115. doi: 10.1016/j.chom.2012.01.009 22341460 PMC3285254

[B78] Schneider J. S. Sklar J. G. Glickman M. S. (2014). The Rip1 protease of *Mycobacterium tuberculosis* controls the SigD regulon. J. Bacteriol 196, 2638–2645. doi: 10.1128/JB.01537-14 24816608 PMC4097585

[B79] Serafini A. Pisu D. Palù G. Rodriguez G. M. Manganelli R. (2013). The ESX-3 secretion system is necessary for iron and zinc homeostasis in *Mycobacterium tuberculosis* . PloS One 8, e78351. doi: 10.1371/journal.pone.0078351 24155985 PMC3796483

[B80] Shafeeq S. Kuipers O. P. Kloosterman T. G. (2013). The role of zinc in the interplay between pathogenic streptococci and their hosts. Mol. Microbiol. 88, 1047–1057. doi: 10.1111/mmi.12256 23650945

[B81] Sharma K. K. Singh D. Mohite S. V. Williamson P. R. Kennedy J. F. (2023). Metal manipulators and regulators in human pathogens: A comprehensive review on microbial redox copper metalloenzymes "multicopper oxidases and superoxide dismutases". Int. J. Biol. Macromol 233, 123534. doi: 10.1016/j.ijbiomac.2023.123534 36740121

[B82] Shi X. Festa R. A. Ioerger T. R. Butler-Wu S. Sacchettini J. C. Darwin K. H.. (2014). The copper-responsive RicR regulon contributes to *Mycobacterium tuberculosis* virulence. mBio 5:e00876-13. doi: 10.1128/mBio.00876-13 24549843 PMC3944814

[B83] Shyam M. Shilkar D. Rakshit G. Jayaprakash V. (2022). Approaches for targeting the mycobactin biosynthesis pathway for novel anti-tubercular drug discovery: where we stand. Expert Opin. Drug Discovery 17, 699–715. doi: 10.1080/17460441.2022.2077328 35575503

[B84] Siegrist M. S. Unnikrishnan M. McConnell M. J. Borowsky M. Cheng T. Y. Siddiqi N.. (2009). Mycobacterial Esx-3 is required for mycobactin-mediated iron acquisition. Proc. Natl. Acad. Sci. U.S.A. 106, 18792–18797. doi: 10.1073/pnas.0900589106 19846780 PMC2774023

[B85] Sivakolundu S. Mannela U. D. Jain S. Srikantam A. Peri S. Pandey S. D.. (2013). Serum iron profile and ELISA-based detection of antibodies against the iron-regulated protein HupB of *Mycobacterium tuberculosis* in TB patients and household contacts in Hyderabad (Andhra Pradesh), India. Trans. R Soc. Trop. Med. Hyg 107, 43–50. doi: 10.1093/trstmh/trs005 23222944

[B86] Spagnuolo M. S. Cigliano L. Maresca B. Pugliese C. R. Abrescia P. (2011). Identification of plasma haptoglobin forms which loosely bind hemoglobin. Biol. Chem. 392, 371–376. doi: 10.1515/bc.2011.033 21294680

[B87] Stafford S. L. Bokil N. J. Achard M. E. Kapetanovic R. Schembri M. A. McEwan A. G.. (2013). Metal ions in macrophage antimicrobial pathways: emerging roles for zinc and copper. Biosci. Rep. 33:e00049. doi: 10.1042/BSR20130014 23738776 PMC3712485

[B88] Stoop E. J. Bitter W. van der Sar A. M. (2012). Tubercle bacilli rely on a type VII army for pathogenicity. Trends Microbiol. 20, 477–484. doi: 10.1016/j.tim.2012.07.001 22858229

[B89] Taranath T. C. Patil B. N. (2016). Limonia acidissima L. leaf mediated synthesis of zinc oxide nanoparticles: A potent tool against *Mycobacterium tuberculosis* . Int. J. Mycobacteriol 5, 197–204. doi: 10.1016/j.ijmyco.2016.03.004 27242232

[B90] Theriault M. E. Pisu D. Wilburn K. M. Lê-Bury G. MacNamara C. W. Michael Petrassi H.. (2022). Iron limitation in *M. tuberculosis* has broad impact on central carbon metabolism. Commun. Biol. 5, 685. doi: 10.1038/s42003-022-03650-z 35810253 PMC9271047

[B91] Tinaztepe E. Wei J. R. Raynowska J. Portal-Celhay C. Thompson V. Philips J. A. (2016). Role of metal-dependent regulation of ESX-3 secretion in intracellular survival of *Mycobacterium tuberculosis* . Infect. Immun. 84, 2255–2263. doi: 10.1128/IAI.00197-16 27245412 PMC4962639

[B92] Wagner D. Maser J. Lai B. Cai Z. Barry C. E. 3rd Höner Zu Bentrup K.. (2005). Elemental analysis of *Mycobacterium avium*-, *Mycobacterium tuberculosis*-, and *Mycobacterium smegmatis*-containing phagosomes indicates pathogen-induced microenvironments within the host cell's endosomal system. J. Immunol. 174, 1491–1500. doi: 10.4049/jimmunol.174.3.1491 15661908

[B93] Waldron K. J. Robinson N. J. (2009). How do bacterial cells ensure that metalloproteins get the correct metal? Nat. Rev. Microbiol. 7, 25–35. doi: 10.1038/nrmicro2057 19079350

[B94] Ward S. K. Hoye E. A. Talaat A. M. (2008). The global responses of Mycobacterium tuberculosis to physiological levels of copper. J Bacteriol 190(8), 2939–2946. doi: 10.1128/jb.01847-07 18263720 PMC2293257

[B95] Ward S. K. Abomoelak B. Hoye E. A. Steinberg H. Talaat A. M. (2010). CtpV: a putative copper exporter required for full virulence of *Mycobacterium tuberculosis* . Mol. Microbiol. 77, 1096–1110. doi: 10.1111/mmi.2010.77.issue-5 20624225 PMC2965804

[B96] Weinberg E. D. (1974). Iron and susceptibility to infectious disease. Science 184, 952–956. doi: 10.1126/science.184.4140.952 4596821

[B97] Weiss G. Schaible U. E. (2015). Macrophage defense mechanisms against intracellular bacteria. Immunol. Rev. 264, 182–203. doi: 10.1111/imr.12266 25703560 PMC4368383

[B98] White C. Kambe T. Fulcher Y. G. Sachdev S. W. Bush A. I. Fritsche K.. (2009). Copper transport into the secretory pathway is regulated by oxygen in macrophages. J. Cell Sci. 122, 1315–1321. doi: 10.1242/jcs.043216 19351718 PMC2671928

[B99] WHO (2023) Global tuberculosis report 2023. Available online at: https://www.who.int/teams/global-tuberculosis-programme/tb-reports.

[B100] Wisedchaisri G. Chou C. J. Wu M. Roach C. Rice A. E. Holmes R. K.. (2007). Crystal structures, metal activation, and DNA-binding properties of two-domain IdeR from *Mycobacterium tuberculosis* . Biochemistry 46, 436–447. doi: 10.1021/bi0609826 17209554

[B101] Wolschendorf F. Ackart D. Shrestha T. B. Hascall-Dove L. Nolan S. Lamichhane G.. (2011). Copper resistance is essential for virulence of *Mycobacterium tuberculosis* . Proc. Natl. Acad. Sci. U.S.A. 108, 1621–1626. doi: 10.1073/pnas.1009261108 21205886 PMC3029754

[B102] Yang L. Hu X. Chai X. Ye Q. Pang J. Li D. (2022). Opportunities for overcoming tuberculosis: Emerging targets and their inhibitors. Drug Discov Today 27(1), 326–336. doi: 10.1016/j.drudis.2021.09.003 34537334

[B103] Yaghubi Kalurazi T. Jafari A. (2021). Evaluation of magnesium oxide and zinc oxide nanoparticles against multi-drug-resistance *Mycobacterium tuberculosis* . Indian J. Tuberc 68, 195–200. doi: 10.1016/j.ijtb.2020.07.032 33845951

[B104] Yeruva V. C. Duggirala S. Lakshmi V. Kolarich D. Altmann F. Sritharan M. (2006). Identification and characterization of a major cell wall-associated iron-regulated envelope protein (Irep-28) in *Mycobacterium tuberculosis* . Clin. Vaccine Immunol. 13, 1137–1142. doi: 10.1128/CVI.00125-06 17028216 PMC1595321

[B105] Zackular J. P. Chazin W. J. Skaar E. P. (2015). Nutritional immunity: S100 proteins at the host-pathogen interface. J. Biol. Chem. 290, 18991–18998. doi: 10.1074/jbc.R115.645085 26055713 PMC4521021

[B106] Zondervan N. A. van Dam J. C. J. Schaap P. J. Martins Dos Santos V. A. P. Suarez-Diez M. (2018). Regulation of three virulence strategies of *Mycobacterium tuberculosis*: A success story. Int. J. Mol. Sci. 19. doi: 10.3390/ijms19020347 PMC5855569 29364195

