# Supplementary material for: The role of transcriptional regulators in metal ion homeostasis of Mycobacterium tuberculosis
Source: Front Cell Infect Microbiol. 2024 Mar 11;14:1360880. doi: 10.3389/fcimb.2024.1360880 (PMC10961391; doi:10.3389/fcimb.2024.1360880)
Supplement: Supplementary file 1 [file Table_1.pdf]

Table S1 Summary of the metal-dependent transcriptional regulators

Members	Name of TF	Functions	Sensed metals	References
Fur	FurA（Rv1909c）	Regulated the expression of genes associated with stress response and intracellular survival, while showing no impact on genes related to the dynamic homeostasis of metals. Compared with the wild-type strain, the primary role of FurA in MAP is to enhance intracellular survival in macrophages by regulating oxidative stress responses.	None	(Eckelt et al., 2015; Liu et al., 2019)
	FurB/Zur (Rv2359)	Regulates the expression of several genes related to Zn ion uptake	Zn^{2+}	(Canneva et al., 2005; Lucarelli et al., 2007; Maciag et al., 2007)
DtxR	IdeR(Rv2711)	Repress the expression of iron uptake genes when the intracellular iron concentration is enriched, thus preventing the iron concentration from becoming too high to reach toxic levels. Regulates mycobacteriocin synthesis by repressing the transcription of genes associated with mycobacteriocin synthesis in the presence of iron. survival of the IdeR-deficient strains was compromised in macrophage and mouse models, underscoring the significant contribution of IdeR to the iron homeostasis, growth and virulence of MTB	Fe^{2+}, Zn^{2+}	(Pandey and Rodriguez, 2014; Bradley et al., 2020).

	MntR/SirR (Rv2788)	Manganese-binding transcriptional regulator	Mn^{2+}	(Yan et al., 2017)
MerR	ZntR(Rv3334）	Probable transcriptional regulatory protein	Zn^{2+}	(Mikhaylina et al., 2018)
	Rv2034	Transcriptional regulator involved in the regulation of lipid metabolism and hypoxic response, regulator of *phoP* and *groEL2*	Zn^{2+}, Cd^{2+}	(Gao et al., 2011)
	KmtR (Rv0827c)	Metal sensor transcriptional regulator	Ni^{2+}, Co^{2+}	(Campbell et al., 2007)
ArsR-SmtB	CmtR (Rv1994c)	Metal sensor transcriptional regulator	Cd^{2+}, Pb^{2+}	(Chauhan et al., 2009)
	SmtB(Rv2358)	Metal transcriptional regulatory protein	Zn^{2+}	(Canneva et al., 2005)
	NmtR(Rv3744)	Metal sensor transcriptional regulator	Ni^{2+}, Co^{2+}	(Campbell et al., 2007)
CsoR-RcnR	CsoR(Rv0967)	Regulate the operon containing four genes *(rv0967-rv0970)*, and this regulatory activity is copper-dependent. *Csor (rv0967)* and *CtpV (cv0969)* genes in the *cso* operon are induced by an increasing level of copper ions.	Cu^{+}	(Liu et al., 2007)
	RicR(Rv0190）	A homologue of CsoR, is predicted to share a similar overall structure with CsoR.	Cu^{+}	(Festa et al., 2011)
Others	HupB/MDP1/ LBP(Rv2986c)	Activates the synthesis of iron carriers in MTB. The HupB-complemented strain exhibited a similar level of iron carrier	Fe^{3+}	(Pandey et al., 2014;

	production compared to that of the wild type. HupB promotes the expression of MB and cMBT, and it induces the Th2 immune response. Mutant strains lacking HupB fail to survive in macrophages, highlighting the crucial role of HupB in the host interactions.		Choudhury et al., 2022)
Rv1474	Rv1474c orchestrates the iron-dependent regulation of Acn *in vivo,* when MTB is grown under iron-deficient conditions.	Fe^{2+}	(Balakrishnan et al., 2017)
SigC(Rv2069)	Preventing copper starvation and serves as a transcriptional regulator of copper ion acquisition when copper is limited, the growth of *sigC*-deficient strains was slower than that of the wild-type strain in a copper ion-deprived medium.	Cu^{2+}	(Grosse-Siestrup et al., 2021)
Rv0474	Copper-responsive transcriptional regulator	Cu^{2+}	(Raghunandanan et al., 2018)
